# Effectiveness and safety of tendon-bone-setting in postpartum women with sacroiliac joint pain: A retrospective multicenter study

**DOI:** 10.1097/MD.0000000000036858

**Published:** 2024-01-19

**Authors:** Min Long, Yunan Dong, Huixian Liu

**Affiliations:** a Women Healthcare Centre, Shenzhen Maternal and Child Healthcare Hospital, Futian District, Shenzhen, Guangdong, China; b Women’s Health Division, Shenzhen Luohu District Maternal and Child Health Care Hospital, Luohu District, Shenzhen, Guangdong, China.

**Keywords:** effectiveness, postpartum sacroiliac joint pain, safety, tendon-bone-setting

## Abstract

Sacroiliac joint (SIJ) pain leads to abnormal joint loading, and is a key risk factor for joint degeneration. This study aimed to determine the effect of tendon-bone-setting on postpartum women with SIJ pain. Multicenter retrospective review of medical records and electroencephalography reports in 10 academic medical centers. 328 postpartum women with sacroiliac joint pain were divided into 2 groups according to the methods of therapy. Group (A) (n = 203) received acupuncture combined with tendon-bone-setting for twenty days, whereas group (B) (n = 125) received only the same acupuncture for twenty days. The outcome measures were the mean values of numeric pain rating scale (NPRS), present pain intensity (PPI) scale, visual analog scale (VAS) and Japanese orthopedic association (JOA) score to evaluate pain intensity, oswestry disability index (ODI), quebec back pain disability scale (QBPDS), active straight leg raise (ASLR) and back pain function scale (BPFS) to evaluate the functional disability, pressure pain thresholds (PPT) at 5 chosen points in the sacroiliac joint region to assess pain sensitivity. All of them were evaluated before and after treatment. The effectiveness from short to long term, as well as safety was assessed in this study. A comparison of the 2 groups after treatment showed statistically significant increases in the mean values of BPFS, JOA and PPT at the 5 chosen points (*P* < .05), as well as significant reductions in the scores of QBPDS, ODI, ASLR, NPRS, VAS and PPI (*P* < .05) in favor of group (B). In addition, after treatment for 2 weeks, the considered effective rate in the group (A) was significantly higher than that in the group (B) (*P* < .05). Also, the cumulative incidence of pain relief at 24 months in the group (A) was greater compared with the group (B) as determined by Kaplan–Meier analysis (*P* < .05). Interestingly, none serious adverse event for the participants was reported. Tendon-Bone-Setting is effective and safe in treating sacroiliac joint pain for the postpartum women patients in the short and long terms through decreasing pain sensitivity and intensity, as well as improving functional ability.

## 1. Introduction

The sacroiliac joint (SIJ), the largest synovial joint in the body, is the tough and fibrous joints, with some limited but important movement, that translates the forces from the spine to the pelvis and legs.^[1]^ Besides, SIJ is one of the most common sources of chronic low back pain (LBP), because it is a highly specialized joint with stability, limited flexibility and upper body support.^[2]^ SIJ pain is the third most common cause of chronic lower back pain, accounting for approximately 10% to 15% of cases^.[3]^ It was reported that, SIJ pain affects individuals with chronic, nonradicular pain and the predisposing factors for it include true and apparent leg length discrepancy, older age, inflammatory arthritis, previous spine surgery and trauma.^[4]^

Pregnancy is the most important stage in every woman life, where many physiological changes occur that cause the child to be carried in the womb.^[5]^ During pregnancy, a woman generally gains weight to about 20 and 40 pounds, which moves the body center of gravity forward, increasing the moment arm of the force applied to the lumbar spine^.[6]^ Interestingly, there are lot of modifications that take place within the female body during pregnancy for the growth of the fetus,^[7]^ which showed that pregnancy was also an important trigger for SIJ pain. In pregnancy, the hormonal changes lead to increased laxity in the ligaments which is what is thought to make SIJ pain more common in this patient population.

Postpartum is a duration that start straight away after delivery and ends at almost month and a half. During pregnancy and after childbirth, the alignment of the bones, especially around the pelvis, changes considerably.^[8]^ Postpartum SIJ pain is a kind of aseptic inflammation caused by tissue changes of the SIJ such increased pelvic asymmetry after delivery, such as congestion, edema, adhesion, which can cause persistent low back pain and lower limb dysfunction.^[9]^ The SIJ pain due to tissue injury accounts for approximately 76.7%.^[10]^ A recent study from Haq K et al demonstrated that the prevalence of SIJ pain in postpartum women is 66.4% and the pain was associated with duration, course and sensation, which have affected their daily routine due to the negligence.^[11]^ Also, SIJ pain places a greater load on the unaffected leg and increases the hip moment on the affected side. The result of these alterations may lead to abnormal joint loading, which is a key risk factor for joint degeneration.^[12]^ Therefore, early and timely treatment of SIJ pain is of great clinical importance.

To relieve SIJ pain, several techniques have been reported. Among them, physical therapy approaches confirmed manual improvement of SIJ malalignment by reestablishing the stability of the lumbar spine and pelvic muscles.^[13]^ Nevertheless, the results of subsequent management of SIJ pain are limited, necessitating additional investigation of several operational procedures for comparison. Various physical therapies, such as tendon-bone-setting is frequently used in physical therapy practice.^[14]^ In this study, it was aimed to detect the impact of tendon-bone-setting on postpartum SIJ pain.

## 2. Patients and methods

### 2.1. Study design

This was a retrospective multicenter study, which was approved by the institutional review board of each center (number for Shenzhen Maternal and Child Healthcare Hospital: 20201026A; number for Shenzhen Luohu District Maternal and Child Health Care Hospital: 20201101A). Also, it was conducted in accordance with the Declaration of Helsinki and the 2017 Ethical Guidelines for Medical and Health Research Involving Human Subjects in Japan. The medical records of consecutive postpartum patients with SIJ pain treated with tendon-bone-setting from January 2018 to October 2020 were retrospectively reviewed including demographic data, pharmacy records, clinical characteristics, comorbidities, at al. Written informed consent was waived because of the retrospective design of this study and information on the right to opt out of the study was presented. Methods of identification were specific to each institution in order to minimize recollection bias.

### 2.2. Participants

The classification of postpartum patients with SIJ pain for enrollment of this study was retrospectively assessed using the following inclusion criteria for SIJ pain: to have unilateral or bilateral pain in back or sacral area (positive posterior pain provocation test for sacroiliac joint dysfunction), to be 20 to 35 years old and to have a body mass index (BMI) that ranged between (25–30) kg/m^2^, to be referred from orthopedist after examination within 6 to16 weeks since last delivery, to be sedentary, ambulatory, nonsmoking women who had reached natural menopause at least 1 year preceding the study and had no history of bilateral surgical removal of the ovaries and/or the uterus. The exclusion criteria were as follows, had any surgery, infection, deformity, tumor and/or fracture in the lumbar vertebrae region or any heart disease, had acute pelvic bacterial or viral infections or leg length discrepancy, had spinal or hip joint disease or a positive straight leg raising test, (4) received nonsteroidal anti-inflammatory drugs, hormonal therapy, or corticosteroid injections. A detailed medical and gynecological history was taken from each patient, including the number of deliveries from (1–3) time, age, weight and history of neurological disorder.

### 2.3. Procedure information

**Figure 1.**
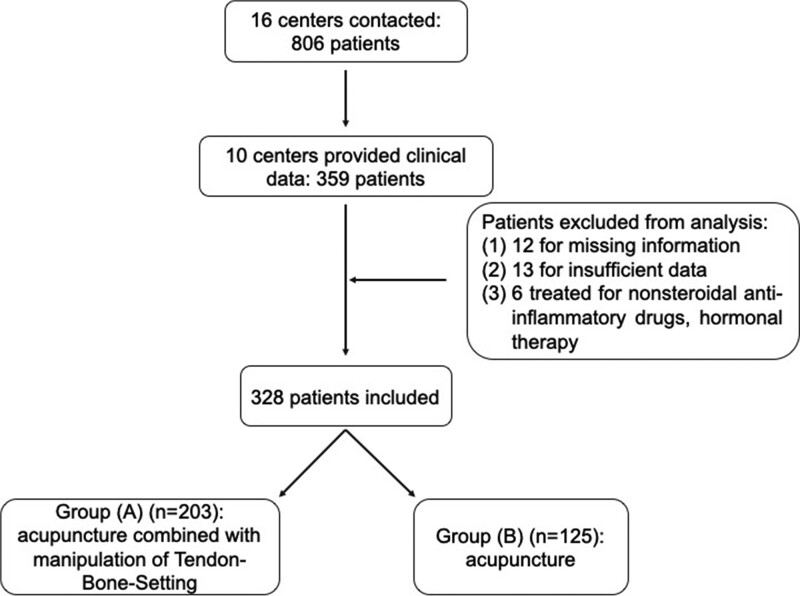
Flow of participants in this study. 806 postpartum women with SIJ pain received treatments including tendon-bone-setting and acupuncture from January 2018 to October 2020. A positive response to requests for clinical data for patients was obtained for only 328 patients, then 31 patients were excluded from the analysis.

According to the methods of therapy, the patients were divided into the following 2 groups: Group (A) (study group) and Group (B) (control group). The patients in the Group (A) (n = 203) were received acupuncture combined with tendon-bone-setting for treatment, while those patients in the Group (B) (n = 125) were only treated with acupuncture. The flow of participants in this study could be seen in Figure 1.

1. Acupuncture: Local acupuncture points were selected individually after diagnostic palpation to identify sensitive spots (Shang Liao, Shen Yu, Wei Zhong, Guan Yuan Yu, Ju Liao, Pang Guan Yu).^[15]^ The patient was placed prone on the treatment bed, with the affected lower limb and lumbosacral area exposed, and the points were routinely disinfected. The needles (Suzhou medical supplies factory Co. Ltd. Suzhou, China) were made of stainless steel (0.14 mm × 40 mm) and inserted intramuscularly to a depth of 15 to 70 mm to evoke needle sensation, described as tension, numbness, and often a radiating sensation from the point of insertion, reflecting activation of muscle-nerve afferents. The needles were left in situ for 30 minutes and manually stimulated every 10 minutes. Treatment was given once every 2 days for over twenty days.
2. Tendon-bone-setting: The manufacturers’ approach was based on the existing methods.^[14]^ Firstly, the patient was placed in the supine position, with both hands being crossed on the abdomen and the affected limb being bent at the hip and knee. The operator held the patient ankle and knee with both hands, and rotated the patient from inside to outside 7 times followed by sending one inch of force to the opposite shoulder. After that, the patient was placed in the lateral position, with the affected limb being on top. The patient remained relaxed, and then bended the knee and hip, with both hands being crossed on the chest. The operator put one hand on the patient shoulder, and the other hand was placed on the patient sciatic tuberosity on the affected side. Then, the operator reversed the force, and rotated the spine by one inch of force. Secondly, the patient was placed in prone position and kept relaxed, then the operator stood on the patient affected side, and one hand was placed on the sacrum of the patient sacroiliac joint, while the other hand was placed on the patient knee, and then the reverse force was applied to the maximum extension to reset by one-inch force.

### 2.4. Outcome measures

Before treatment and after all participants were assessed to complete a series of treatment, the main outcome measurement variables including evaluation of pain intensity and assessment of functional disability were as following:

1. Measurement of functional disability solely in terms of activity by the quebec back pain disability scale (QBPDS)^[16]^: the 20 items are answered on a 6-point numerical rating scale indicating the level of difficulty (0, “not difficult at all”; 1, “minimally difficult”; 2, “somewhat difficult”; 3, “fairly difficult”; 4, “very difficult”; and 5, “unable to do”). The total score is the sum of all the items, and ranges from 0 to 100 points.
2. Appraisal of s the level of LBP pain using the oswestry disability index (ODI)^[17]^: the ODI contains 10 items on the degree of severity to which back (or leg) problems have affected the ability to manage activities in everyday life. Each item is scored on a 6-point scale (0–5), with “‘0’” representing “‘no limitation’” and “‘5’” representing “‘maximal limitation’.” The ODI score is calculated by the following formula: [total score/(5 × number of questions answered)]×100. The range of the score is from 0 to 100. A higher score indicates more functional limitation due to back problems.
3. Patients function level by using back pain function scale (BPFS)^[18]^: it consists of 12 objects of daily activities with 5 different answers. Patient response to each item was reported by each participant conferring to her capability to apply this activity. Inability to perform any activity referred to zero score, while no difficulty in any activity referred to the maximum, which was 60 score.
4. Estimation of the ability to transfer load from the legs to the trunk by active straight leg raise (ASLR) test^[19]^: the test was performed according to the instruction (try to raise your legs, one after the other, above the couch for 20 cm without bending the knee), and impairment in raising 1 leg was scored on a 6-point scale from 0 to 5 (not difficult at all = 0; unable to do = 5). The scores of both sides were added, so that the summed score ranged from 0 to 10.
5. Measurement of the patient level of pain by the numeric pain rating scale (NPRS)^[20]^: an 11-point NPRS ranging from 0 to10 was anchored on the left with the phrase “No Pain” and on the right with the phrase “Worst Imaginable Pain.” Patients rated their current, best, and worst level of pain during the last 24 hours. The average of the 3 ratings was used to represent the patient overall pain intensity.
6. Sacroiliac pain via using visual analogue scale (VAS)^[21]:^ the VAS for disability consisted of 3 100 mm lines, each labeled at the left end as “no disability” (0 mm) and at the right end as “very severe disability” (100 mm). Patients were asked to draw a vertical mark on each line, one on the upper line for their current disability, one on the middle line for the disability at their best (least disabled) period during the previous week and one on the lower line for the disability at their worst (most disabled) period during the previous week.
7. Measurement of pain intensity by present pain intensity (PPI) scale^[22]^: it was used for assessing pain severity in clinical practice, which was scored from a single question of 6 pain descriptors as being: no pain = 0, mild pain = 1, moderate pain = 2, severe pain = 3, unbearable pain = 4.
8. Assessment of functionality and pain through Japanese orthopedic association (JOA) score^[23]^: the JOA score assesses functionality and pain and contains 4 sections (14 items). Overall score on the questionnaire ranges from −6 to 29, with higher scores indicating better conditions.
9. Assessment of patient pain sensitivity by pressure pain thresholds (PPT) at 5 selected points in the sacroiliac joint region of the affected side using a pressure algomete with a probe area of 1 cm^2^, through the application of a constant pressure axially on each point until the pain was reported by the participant. The algometry could measure 10 kg/cm^2^ and be accurate to 0.1 kg/cm^2^. Each point was measured 3 times with a ten-second interval between them; the mean of them was then calculated for each point to be utilized for statistical analysis.^[24]^
10. To evaluate the pain after treatment, we used 6 of the 7 motions of the “restriction of activities of daily living” in the assessment of treatment for low back pain by the JOA scoring system.^[25]^ The restriction of those motions was assessed as follows: 0, severe restriction; 1, moderate restriction; 2, no restriction; 0.5, severe to moderate restriction; 1.5, moderate to no restriction. The pain assessment was represented by the sum of those scores, with a full score being 12 points. The treatment was defined as effective when the score was 10.5 points or higher after treatment because those who had higher scores were satisfied with the treatment. The improvement rate was also calculated as follows, (Score after treatment—score before treatment)/(12—score before treatment) × 100%.
11. Safety assessments: after the treatment through the follow-up visit, treatment-emergent adverse events were defined according to the International Conference for Harmonization Note for Guidance on Clinical Safety Data Management.^[26]^

### 2.5. Statistical analysis

Data of continuous variables were shown as mean ± standard deviation (SD) with Student *t* test, while categorical variables were expressed with percentage using Chi-squared test (or Fisher exact test if necessary) for univariate comparison. Kaplan–Meier survival analysis with the log-rank test was performed to compare the cumulative incidence of sustained pain relief between the 2 groups. All statistical analyses were performed using SPSS 25.0 software (Chicago, IL). A 2-sided *P* < .05 was deemed to indicate statistical significance.

## 3. Results

### 3.1. Population characteristics

328 subjects were included in this study. Baseline characteristics are outlined in Table 1, which presents the overall information of both groups (A and B) at the start of the study. There were no statistically significant differences between these 2 patients in terms of age, BMI, number of pregnancies and parity, time since last delivery, total gestational weight gain, and status of smoking (*P* > .05).

**Table 1:** Baseline characteristics of subjects.

		Group (A) (n = 203)	Group (B) (n = 125)	t/χ^2^ value	*P* value
Age (yr)		32.21 ± 5.11	33.03 ± 5.46	1.375	.170
BMI (kg/m^2^)		25.65 ± 5.33	26.03 ± 4.74	0.634	.514
Education (yr)		17.04 ± 2.12	16.86 ± 3.43	0.586	.557
Number of pregnancies		2.14 ± 0.39	2.09 ± 0.57	0.942	.347
Number of parity		1.65 ± 0.36	1.71 ± 0.45	1.330	.184
Time since last delivery (wk)		13.14 ± 5.74	14.09 ± 4.56	1.570	.117
Total gestational weight gain (kg)		16.32 ± 4.56	17.16 ± 5.33	1.518	.130
Smoking	Yes	65	49	1.457	.227
	No	138	76		

BMI = body mass index.

### 3.2. Descriptive statistics for QBPDS, ODI, BPFS, and ASLR in both groups at different periods

Table 2 represent within and between groups (A and B) comparison mean QBPDS, ODI, BPFS and ASLR pre and post treatment. Before treatment, there was no significant difference between both groups (A and B) (*P* > .05). Comparison between the group A and B post treatment showed a significant decrease in QBPDS, ODI and ASLR of group A compared with that of group B (*P* < .05), while the BPFS in the group (A) was increased than the group B (*P* < .05).

**Table 2:** Descriptive statistics for QBPDS, ODI, BPFS, and ASLR in both groups at different periods (±s)

Group	QBPDS		ODI		BPFS		ASLR	
	Pre	Post	Pre	Post	Pre	Post	Pre	Post
Group (A) (n = 203)	42.32 ± 8.76	15.09 ± 4.12	66.45 ± 8.76	32.75 ± 8.63	20.43 ± 4.54	51.09 ± 5.65	6.57 ± 0.79	2.83 ± 0.51
Group (B) (n = 125)	40.76 ± 9.92	20.15 ± 5.25	65.68 ± 9.16	44.53 ± 9.86	21.06 ± 4.93	44.65 ± 5.01	6.49 ± 0.68	3.57 ± 0.72
t value	1.488	9.712	0.760	11.364	1.181	10.460	0.938	10.873
*P* value	.138	.000	.448	.000	.239	.000	.349	.000

ASLR = active straight leg raise, BPFS = back pain function scale, ODI = oswestry disability index, QBPDS = quebec back pain disability scale.

### 3.3. Descriptive statistics for NPRS, VAS, PPI, and JOA in both groups at different periods

As shown in Table 3, Student *t* test was used to compare between the median values of NPRS, ODI, PPI, and BPFS in both groups (A and B). Before the treatment, there was no statistical significant difference between group A and group B as *P* > .05. On the other hand, after treatment findings recorded a significant decrease in the median values of NPRS, VAS, and PPI in group A when compared with its corresponding values in group B as *P* value was <.05. In addition, the score of improvement in JOA was higher in group A than that in group B, and the difference was greatly significant (*P* < .05).

**Table 3:** Descriptive statistics for NPRS, VAS, PPI, and JOA in both groups at different periods (±s)

Group	NPRS		VAS		PPI		JOA	
	Pre	Post	Pre	Post	Pre	Post	Pre	Post
Group (A) (n = 203)	6.45 ± 0.92	3.43 ± 0.57	3.53 ± 0.53	1.03 ± 0.58	2.51 ± 0.42	0.82 ± 0.33	17.32 ± 3.41	28.46 ± 3.07
Group (B) (n = 125)	6.38 ± 0.99	4.25 ± 0.71	3.51 ± 0.41	1.62 ± 0.59	2.47 ± 0.38	1.09 ± 0.21	17.53 ± 3.13	24.78 ± 3.49
t value	0.650	11.504	0.361	8.889	0.868	8.182	0.559	10.002
*P* value	.516	.000	.719	.000	.386	.000	.577	.000

JOA = Japanese orthopedic association, NPRS = numeric pain rating scale, PPI = present pain intensity, VAS = visual analog scale.

### 3.4. Descriptive statistics for PPT at the 5 chosen points, in both groups at different periods

On the basis of Table 4, no highly significant differences were observed in term of the mean values of PPT at the 5 chosen points (point I, point II, point III, point IV, and point V) within both groups (*P* > .05). Comparing both groups after treatment revealed statistically significant increases in the mean values of PPT at the 5 chosen points in favor of group (B) (*P* < .05).

**Table 4:** Descriptive statistics for PPT at the 5 chosen points in both groups at different periods (±s, kg/cm^2^)

		Group (A) (n = 203)	Group (B) (n = 125)	t value	*P* value
At point I	Pre	7.85 ± 1.38	8.01 ± 1.45	1.000	.318
	Post	13.23 ± 2.24	11.09 ± 1.97	8.790	.000
At point II	Pre	7.97 ± 1.29	8.12 ± 1.24	1.038	.300
	Post	12.98 ± 2.10	10.58 ± 1.08	11.845	.000
At point III	Pre	7.71 ± 1.01	7.87 ± 1.12	1.336	.182
	Post	12.26 ± 1.85	11.02 ± 0.93	6.968	.000
At point IV	Pre	7.42 ± 1.12	7.53 ± 1.04	0.887	.376
	Post	12.55 ± 1.23	11.41 ± 0.92	8.935	.000
At point V	Pre	7.58 ± 1.43	7.66 ± 1.25	0.516	.606
	Post	12.87 ± 1.59	11.95 ± 1.27	5.481	.000

PPT = pressure pain thresholds.

### 3.5. Evaluation of the treatment effect in both 2 groups

#### 3.5.1. Effectiveness at 2 weeks’ follow-up.

After treatment for 2 weeks, the considered effective rate was 92.61% (188/203) in the group (A), whereas the effective rate in the group (B) was 45.60% (57/125). In addition, the difference between these 2 groups was greatly significant (*P* < .05).

#### 3.5.2. Long-term follow-up data.

**Figure 2.**
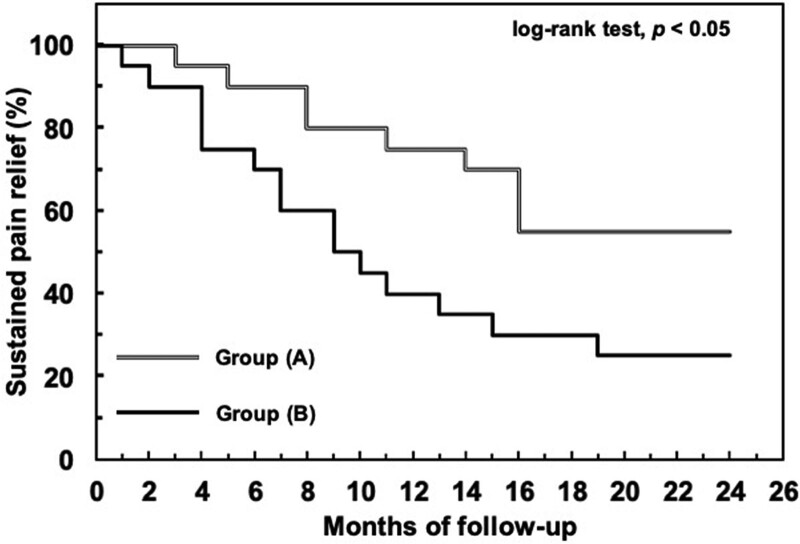
Kaplan–Meier plot showing cumulative incidence of sustained 50% or greater relief of pain, which was significantly higher in the group (A) compared with the group (B), as analyzed by log-rank test, *P* < .05 between these groups.

The cumulative incidence of sustained pain relief at 24 months was 55.3% (95% confidence interval [CI] 37.5%–76.3%) in the group (A) and 25.3% (95% CI 10.7%–37.4%) in the group (B); there was a significant difference between the 2 survival curves (*P* < .05; Figure 2).

### 3.6. Safety assessment in both 2 groups after treatment

Transient aggravation of hip pain lasting 5 days without any intervention other than simple analgesics was reported in some patients without significant difference between groups. None of the participants reported serious adverse event such as long-lasting exacerbation of pain, numbness or weakness, or signs of skin infection.

## 4. Discussion

The SIJ is a true diarthrodial joint, consisting of 2 surfaces held together by fibrous capsule and enjoined with synovial fluid.^[27]^ Postpartum SIJ caused by the change of postpartum pelvic tissue structure and aseptic inflammation such as congestion, edema, and adhesion, resulting in persistent low back pain and lower extremity dysfunction, accounts for about 76.7%.^[28]^ Although there is some variation in reported prevalence rates depending on the sample population and diagnostic criteria, it can be concluded that the SIJ represents a major cause of mechanical LBP in patients of all ages, especially in in women after full-term normal delivery.^[4]^

Treatment for SJP can be conservative, injective, and surgical (fusion).^[29]^ In addition, much of the literature regarding therapeutic options has focused on some alternative treatment approaches to SIJ pain for postpartum women such as radiofrequency denervation, e.g., thermal and cooled radiofrequency.^[28]^ Conservative treatment includes physiotherapy or manipulation (manual therapy, osteopathic treatment, chiropractic adjustments).^[30]^ Physical therapy of conservative management including rest, splint fixation, physiotherapy can provide a viable early option, and there are few reports about conservative treatment with satisfactory results.^[31]^ Manipulation therapy is the advantage and characteristic of traditional Chinese medicine, and it pays special attention to function exercise in the treatment. The purpose of the case report was to describe the impact of tendon-bone-setting on a patient postpartum who met the criteria for SIJ pain based on Laslett cluster of positive SIJ pain provocation signs.

In the present study, we did an experimental study, 328 women between the age group of 22 to 43 years were retrospectively divided into 2 groups (Group A and Group B) according to the methods of therapy. We analyzed and compared both the groups after treatment, there was a statistical difference in pre- and post-intervention analysis of both the groups; however, the result showed statistically significant improvement in Group A than Group B. Because Group A receives acupuncture along with tendon-bone-setting, tendon-bone-setting relieves pain as including changes in decreasing pain sensitivity and intensity, as well as improving functional ability. It was also reported the effectiveness of tendon-bone-setting in joint injury.^[14]^ They suggested that tendon-bone-setting could be used as an adjunct to exercises in treatment for postpartum SIJ pain, which also supports findings of our study.

Our study had several limitations: it was a retrospective study with missing data in the patient source, limited to one region in China and data were not exhaustive. Moreover, the patients who took part in this study were probably not representative of the whole population, with a possible greater adherence to guidelines. Finally, because of the retrospective method, we evaluated the efficacy of tendon-bone-setting regarding postpartum SIJ pain with an after/before comparison which induced more bias than a case control comparison. Despite these limitations, this series documents the potential utility and current patterns of use of tendon-bone-setting for the treatment of postpartum sacroiliac joint pain and provides useful preliminary data for planning a prospective trial.

## 5. Conclusion

A multimodal approach of tendon-bone-setting has a positive impact on pain by decreasing pain sensitivity and intensity, as well as improving functional ability in a patient postpartum with SIJ pain. Further research is warranted to develop stronger evidence to identify specific interventions for the treatment of postpartum SIJ pain.

## Acknowledgments

There are no funding sources to declare. Besides, the authors declare that they have no competing interests relevant to this study and the publication thereof. In addition, we wish to thank the editor, the associate editor, and the 2 anonymous reviewers for their helpful comments and suggestions, which have led to an improvement of this article.

## Author contributions

**Conceptualization:** Min Long.

**Data curation:** Min Long, Yunan Dong, Huixian Liu.

**Formal analysis:** Min Long, Yunan Dong, Huixian Liu.

**Investigation:** Min Long, Yunan Dong, Huixian Liu.

**Writing – original draft:** Min Long.

**Writing – review & editing:** Min Long, Yunan Dong.
